# Characteristics and correlation analysis of postprandial free fatty acids and cortisol levels in males after different meals: a clinical trial

**DOI:** 10.3389/fendo.2026.1760358

**Published:** 2026-03-18

**Authors:** Dandan Liu, Peipei Tian, Yilin Hou, Tingxue Zhang, Yamin Lu, Luping Ren, Chao Wang, Guangyao Song

**Affiliations:** 1 Department of Internal Medicine, Hebei Medical University, Shijiazhuang, Hebei, China; 2 Department of Endocrinology, Hebei General Hospital, Shijiazhuang, Hebei, China; 3 Department of Endocrinology, Baoding First Central Hospital, Baoding, Hebei, China; 4 Department of Endocrinology, Cangzhou Central Hospital, Cangzhou, Hebei, China; 5 Department of Nuclear Medicine, Hebei General Hospital, Shijiazhuang, Hebei, China; 6 Hebei Key Laboratory of Metabolic Diseases, Hebei General Hospital, Shijiazhuang, Hebei, China

**Keywords:** blood lipids, cortisol, free fatty acids, meals, postprandial state

## Abstract

**Objective:**

Elevated levels of free fatty acids (FFA) and cortisol are linked to adverse metabolic effects. The changes in FFA and cortisol levels after different meals and their relationship are still not clear.

**Methods:**

Eleven healthy male volunteers were recruited from Hebei General Hospital. Each participant consumed five different test meals, including high-fat meals, fat meals, protein meals, glucose meals, and fructose meals, with a one-week washout period between interventions. All test meals were consumed between 7:00 and 8:00 am. Blood samples were collected before and after each meal to assess blood lipids, blood glucose, insulin, FFA, and cortisol levels.

**Results:**

After consumption of the fat meals, FFA levels showed an initial rise followed by a gradual decline, peaking at 4 hours after the meal, and remaining above baseline at 6 hours (*P* < 0.001). In contrast, following the high-fat, protein, glucose, and fructose meals, FFA levels exhibited an early suppression with a subsequent rebound, reaching the lowest level at 2 hours after the meal. Among these meals, only after the high-fat meal did FFA levels rise above baseline (*P* = 0.003). After the high-fat and fat meals, cortisol levels declined early after the meal and then increased, with the lowest values observed at 4 and 2 hours, respectively. Following the protein and fructose meals, cortisol levels showed a continued decline over time. After the glucose meals, cortisol levels rose for a short time, peaked at 0.5 hours, and then decreased to below baseline value at 3 hours (*P* = 0.034). Significant positive correlation between cortisol and FFA levels were observed at 5 (r=0.610, *P* = 0.046) and 6 (r=0.824, *P* = 0.002) hours after the high-fat meals and at 3 (r=0.632, *P* = 0.037), 4 (r=0.673, *P* = 0.023), and 6 (r= 0.614, *P* = 0.045) hours after the fat meals.

**Conclusions:**

As dietary fat content increases, postprandial FFA level also rises. The changes in FFA levels after consuming high-fat meals and fat meals for breakfast are closely related to the changes in serum cortisol.

**Clinical trial registration:**

https://www.chictr.org.cn, identifier ChiCTR2100048497.

## 1 Introduction

If the amount of fat from the diet exceeds the actual requirement, the concentration of FFA will increase, which will lead to ectopic lipid deposition, insulin resistance, diabetes, hypertension, coronary heart disease, cerebral infarction, sudden death and increased cancer mortality risks (1). Most patients with hypercortisolism will have hypertension and abnormal blood lipids, and often have ectopic lipid deposition, insulin resistance, diabetes, etc. (2, 3). The harms of the two are so similar. So, is there any relationship between FFA and cortisol?

Free fatty acids (FFA) are needed by the body for energy supply during fasting. Their concentration is higher during fasting, and almost all of it comes from the hydrolysis of TG in adipocytes. The concentrations of metabolic products and hormones in the body change rapidly after meals. The release of FFA is inhibited after meals, and the esterification of FFA in adipose tissue and muscles is enhanced, resulting in a decrease in FFA levels. Later, the FFA carried in chylomicrons is released from dietary fat and absorbed and stored by adipocytes, and some FFA escape and enter the plasma FFA pool, thereby causing an increase in FFA levels in the post-meal period (1, 4).

The level of cortisol will increase after meals. The possible mechanism is that food intake can enhance the function of the hypothalamic-pituitary-adrenal axis, and various intestinal-derived signals can increase cortisol secretion, and at the same time, adrenal extracortical cortisol regeneration is mediated (5, 6).

Currently, few studies compare FFA levels after consuming different meals in the same group of participants, and no studies have been found investigating the correlation between FFA and cortisol levels postprandially. In this study, we not only provided healthy participants with high-fat mixed meals but also pure fat, protein, glucose, and fructose meals, aiming to examine changes in FFA and cortisol levels after different meals and their potential correlations.

## 2 Methods

### 2.1 Study participants

In March 2023, 17 male volunteers aged 30-50 years were recruited from Hebei General Hospital. The study was approved by the Ethics Committee of Hebei General Hospital (approval number: 2021-40). And this study was registered in the Chinese Clinical Trial Registry (Registration Number: ChiCTR2100048497, Website: https://www.chictr.org.cn). During the recruitment process, participants were thoroughly briefed about the experiment and were advised to maintain emotional stability to reduce dropout rates and enhance compliance. All volunteers signed informed consent forms.

All volunteers underwent a health examination, which included measurements of height, weight, and blood pressure. Body mass index (BMI) was calculated using the formula: weight/height² (kg/m²). Fasting blood lipid levels, including total cholesterol (TC), triglycerides (TG), high-density lipoprotein cholesterol (HDL-C), and low-density lipoprotein cholesterol (LDL-C), were tested. Additional tests included liver and kidney function, thyroid function, and an oral glucose tolerance test (OGTT). Volunteers also underwent electrocardiograms, abdominal ultrasound, and vascular ultrasound.

Inclusion criteria: Volunteers had to be of Han ethnicity, with a BMI between 18.5-24 kg/m², normal blood pressure (below 140/90 mmHg), normal blood lipid levels (TG < 1.7 mmol/L, TC < 5.2 mmol/L, LDL-C < 3.4 mmol/L) (7), normal OGTT results (fasting blood glucose < 6.1 mmol/L and 2-hour postprandial blood glucose < 7.8 mmol/L) (8), normal liver and kidney function, and normal thyroid function. Electrocardiogram, abdominal ultrasound, and vascular ultrasound results should also be normal.

Exclusion criteria: Volunteers with needle phobia, blood phobia, or those unable to undergo multiple venous blood draws were excluded. Volunteers with a history of food or drug allergies, or intolerance to high-protein or high-fat foods, smokers, heavy drinkers, vegetarians, those who had experienced significant life events (e.g., divorce, unemployment, death of a loved one) in the past six months, those with a weight change exceeding 3 kg in the past 3 months, those taking medications that affect glucose and lipid metabolism, and those with psychiatric disorders, infectious diseases, kidney disease, heart disease, acute or chronic blood diseases, malignant tumors, a history of stroke in the past 3 months, or those with severe infections, injuries, or surgical history were excluded.

After screening, 11 volunteers met the inclusion and exclusion criteria and were enrolled in the study.

### 2.2 Study design

This study was a prospective, randomized, crossover trial. Each subject consumed five test meals. The glucose meal was designed to follow the OGTT procedure and was completed during the health examination, while the remaining four meals were assigned to participants in a random order generated by a computer. Prior to each test, participants were required to undergo a one-week washout period with a regular diet, and smoking, drinking, or vigorous exercise was prohibited during the trial.

Before each trial, participants fasted from food and water after 22:00 the day before. They got up at 6:00 am in the morning on the second day, and fasting blood samples were collected from 7:00 and 8:00 am through a venous puncture. The time when participants began the trial at the hospital differed by no more than 10 minutes each time. After blood collection, participants consumed the test meal within 10 minutes. They were not allowed to eat anything further until the end of the test but were allowed to drink water freely. Blood samples were collected at 1, 2, 3, 4, 5, and 6 hours post-meal for the high-fat, fat, and protein meals. For the glucose meal (OGTT), blood samples were collected at 0.5, 1, 2, and 3 hours post-meal. For the fructose meal, samples were collected at 0.5, 1, 2, 3, and 4 hours post-meal. Each blood sample volume was approximately 5 mL. The samples were centrifuged at 3000 rpm at 4 °C for 10 minutes, and the serum was collected for analysis.

### 2.3 Test meals

- High-fat meal (High-fat mixed meal, HF): The total caloric value of the test meal is 700 kcal, with a macronutrient ratio of fat:protein:carbohydrate at 60%:25%:15%.
- Fat meal: The test meal contains 75g of fat.
- Protein meal: The test meal contains 75g of protein.
- Glucose meal: The test meal contains 75g of glucose.
- Fructose meal: The test meal contains 75g of fructose.

All test meals are prepared as 300 mL aqueous solutions. Fat is provided by Ferrica™ energy-dense medium-chain triglyceride beverage (Fresenius Kabi Pharmaceuticals, Beijing, China) with an energy density of 5.0 kcal/mL. Protein is provided by Protein Supplement™ whey protein powder (Nestlé Health Science, USA). Glucose is provided by 50% glucose injection (Fresenius Kabi Huari Pharmaceutical Co., Ltd., China). Fructose is provided by crystalline fructose (Xiwang Sugar Industry Co., Ltd., Shandong, China). The formulas for the test meals are detailed in **Table 1**.

**Table 1:** Formula of test meal.

Group	Total calories (kcal)	Fat (g)	Saturated fatty acids (g)	Medium-chain triglycerides (g)	Monounsaturated fatty acids (g)	Polyunsaturated fatty acids (g)	Protein (g)	Carbohydrates (g)
High-fat meal	700	46.7	14.5	12.1	21.4	10.9	43.8	26.3
Fat meal	697.4	75	23.3	19.4	34.3	17.4	-	5.6
Protein meal	323.3	0.9	-	-	-	-	75	3.8
Glucose meal	300	-	-	-	-	-	-	75
Fructose meal	300	-	-	-	-	-	-	75

### 2.4 Detection indicators

The TG, TC, HDL-C, LDL-C, and blood glucose were measured using a fully automated biochemical analyzer (Hitachi 7600, Hitachi, Japan). Insulin was measured by electrochemiluminescence (Roche Cobas e601, Roche, Germany). Cortisol was measured by chemiluminescence immunoassay (MAGLUMI 4000 PLUS, Xind Industries Biomedical Engineering Co., China). FFA was measured using the microplate method (fully automated enzyme-linked immunosorbent assay, Molecular Devices, USA). The laboratory tests were conducted using a blind method.

### 2.5 Statistical methods

Data were analyzed using SPSS 27.0 software. Normally distributed continuous variables were expressed as mean ± standard deviation ( $\bar{\text{x}}$ ± s), while non-normally distributed data were expressed as median and interquartile range (IQR). For normally distributed data with equal variances, independent sample t-tests were used for comparison between two groups, and one-way analysis of variance (ANOVA) was used for comparisons between three or more groups. Repeated measures ANOVA was used to compare differences at different time points for the same indicator and between different meals at the same time. For non-normally distributed data, non-parametric tests were used. To control the risk of false positive results caused by multiple comparisons, when the differences between three or more groups of data are statistically significant, the Bonferroni method is used for pairwise comparisons to correct the results. The mixed-effects model was used to compare the cortisol levels after consuming high-fat meals and fat meals. A one-way linear regression analysis was performed to assess the correlation between cortisol and FFA levels at the same time point after different meals. A P value < 0.05 was considered statistically significant. Graphs were created using GraphPad Prism 9 software.

## 3 Results

### 3.1 Basic data

A total of 11 male participants were included in the study, with an average age of 37.45 ± 5.07 years. Mean systolic and diastolic blood pressure were 116 ± 5 mmHg, and 77 ± 5 mmHg, respectively. The average body mass index (BMI) was 22.33 ± 1.63 kg/m², with a hip circumference of 95.72 ± 3.17 cm, and a waist circumference of 81.82 ± 5.84 cm.

### 3.2 Blood glucose and insulin levels after different meals in participants

**Figure 1:**
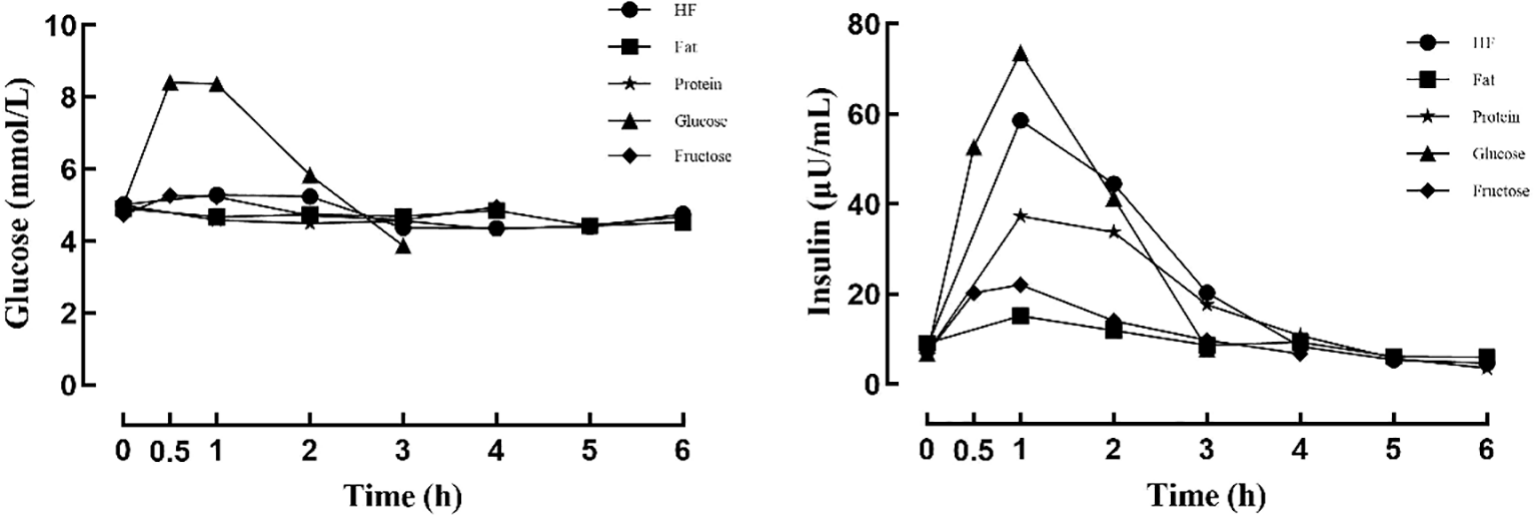
Changes in blood glucose and insulin levels after different meals.

Before consumption of the test meals, no statistically significant differences were observed in glucose or insulin levels among the participants (*P*s > 0.05). After the glucose meal, blood glucose levels showed the largest increase, peaking at 0.5 hours (*P* < 0.05) and then gradually declining. At 3 hours (*P* < 0.05) after meal intake, glucose levels fell below baseline, indicating normal glucose metabolism. In contrast, blood glucose levels after the high-fat, fat, protein, and fructose meals remained within the normal range, with relatively small fluctuations (see **Figure 1**; **Table 2**).

**Table 2:** Changes in blood glucose levels after different meals.

Glucose	High fat	Fat	Protein	Glucose	Fructose	F	*P*
0h	5.02 ± 0.55	4.92 ± 0.32	5.01 ± 0.64	5.04 ± 0.27	4.73 ± 0.39	0.961	0.439
0.5h				8.43 ± 0.92^*^	5.27 ± 0.58^*^	75.579	<0.001
1h	5.29 ± 0.70	4.68 ± 0.36	4.59 ± 0.38	8.38 ± 2.35^*^	5.23 ± 0.51^*^	22.757	<0.001
2h	5.25 ± 0.44	4.74 ± 0.40	4.49 ± 0.57	5.84 ± 1.48^#a^	4.72 ± 0.42^#a^	5.810	<0.001
3h	4.37 ± 0.31^*ab^	4.70 ± 0.24^*^	4.57 ± 0.50^*^	3.87 ± 0.57^*#ab^	4.60 ± 0.25^#a^	9.167	<0.001
4h	4.37 ± 0.29^*ab^	4.86 ± 0.12^c^	4.32 ± 0.42^*^		4.96 ± 0.25^c^	19.541	<0.001
5h	4.40 ± 0.27^*ab^	4.43 ± 0.18^*abcd^	4.45 ± 0.35^*^			0.077	0.927
6h	4.77 ± 0.31^*abcde^	4.53 ± 0.24^*d^	4.68 ± 0.27^de^			3.135	0.065
F	12.488	5.784	3.470	31.166	8.872		
*P*	<0.001	<0.001	0.005	<0.001	<0.001		

*P<0.05 versus the 0h glucose; #, P<0.05 versus the 0.5h glucose; a, P<0.05 versus the 1h glucose; b, P<0.05 versus the 2h glucose; c, P<0.05 versus the 3h glucose; d, P<0.05 versus the 4h glucose; e, P<0.05 versus the 5h glucose.

After all five test meals, insulin levels rose during the early phase after eating and then gradually declined, with peak values reached at 1 hour (*P*s < 0.05). The insulin response was greatest after the glucose meal, followed by the high-fat, protein, fructose, and fat meals. In all cases, insulin levels subsequently returned to baseline or fell below baseline levels (see **Figure 1**; **Table 3**).

**Table 3:** Changes in insulin levels after different meals.

Insulin	High fat	Fat	Protein	Glucose	Fructose	F	*P*
0h	8.41 ± 4.42	9.13 ± 3.77	7.08 ± 2.77	6.87 ± 2.73	6.98 ± 2.19	1.680	0.174
0.5h				52.74 ± 22.35^*^	20.35 ± 8.73^*^	21.395	<0.001
1h	58.64 ± 30.49^*^	15.20 ± 8.80^*^	37.34 ± 15.03^*^	73.65 ± 41.94^*^	22.10 ± 13.11^*^	16.173	<0.001
2h	44.58 ± 33.46^*^	11.94 ± 5.42^*^	33.84 ± 17.16^*^	41.33 ± 26.66^*a^	14.1 ± 7.65^*#a^	7.700	<0.001
3h	20.31 ± 11.71^*ab^	8.74 ± 3.91^ab^	17.69 ± 7.91^*ab^	7.85 ± 6.41^#ab^	9.78 ± 6.75^#ab^	7.340	<0.001
4h	8.38 ± 4.09^abc^	9.36 ± 6.66^ab^	10.87 ± 5.55^*abc^		6.81 ± 3.75^#ab^	1.385	0.266
5h	5.43 ± 2.06^*abcd^	6.22 ± 4.22^*abcd^	5.78 ± 4.31^abcd^			0.215	0.808
6h	4.72 ± 2.43^*abcd^	6.04 ± 3.07^*abc^	3.60 ± 1.61^*abcd^			5.782	0.010
F	20.800	10.168	29.934	19.483	15.426		
*P*	<0.001	<0.001	<0.001	<0.001	<0.001		

*P<0.05 versus the 0h insulin; #:P<0.05 versus the 0.5h insulin; a, P<0.05 versus the 1h insulin; b, P<0.05 versus the 2h insulin; c, P<0.05 versus the 3h insulin; d, P<0.05 versus the 4h insulin; e, P<0.05 versus the 5h insulin.

### 3.3 Blood lipid changes after different meals in participants

**Figure 2:**
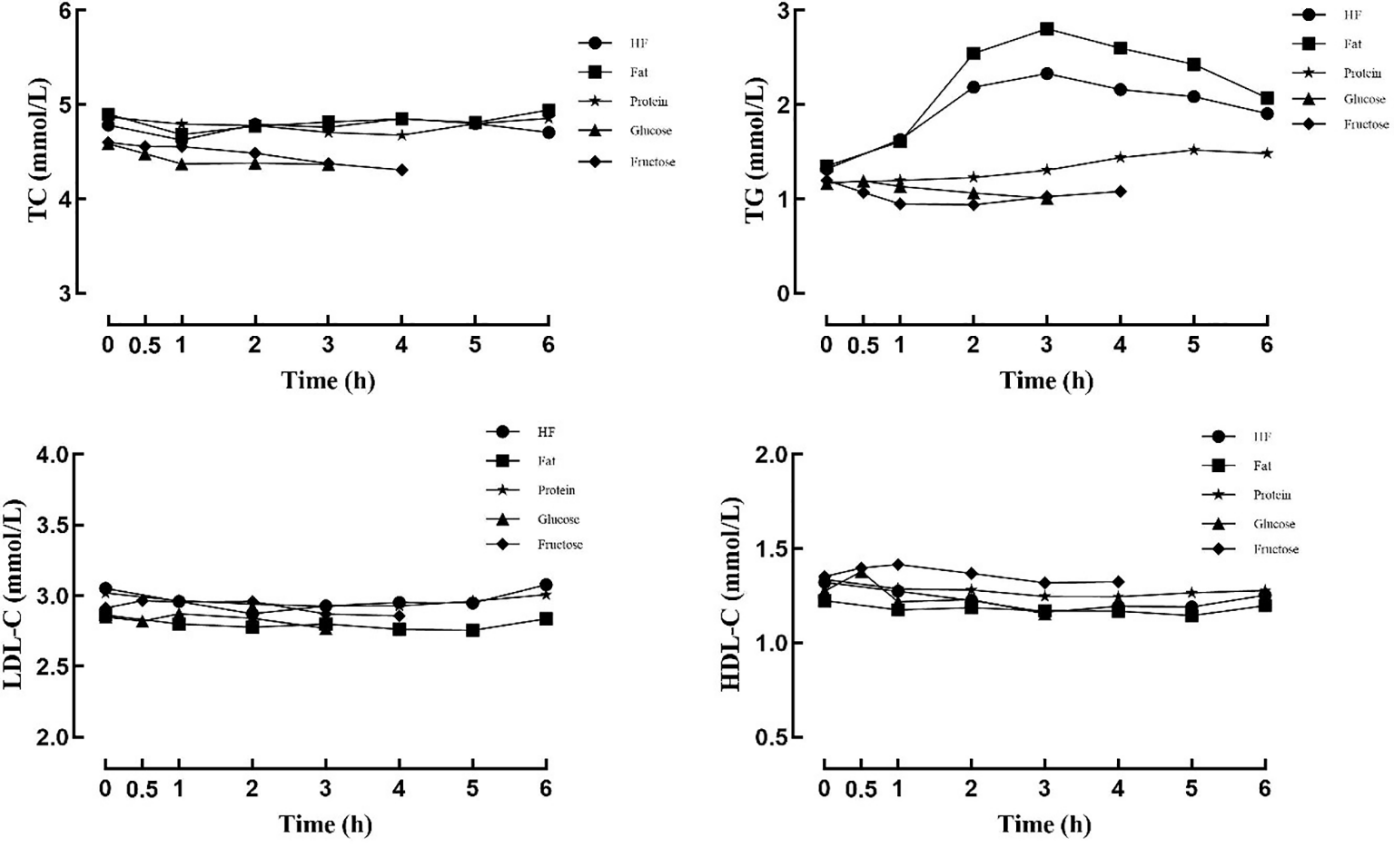
Changes in blood lipids levels after different meals.

Before meal intake, there were no statistically significant differences in total cholesterol, triglycerides, low-density lipoprotein cholesterol, or high-density lipoprotein cholesterol levels among the different meal conditions (*P*s > 0.05). Postprandial TG levels varied considerably depending on meal type. After the high-fat and fat meals, TG concentrations rose during the early period, reached a peak at 3 hours, and remained above baseline at 6 hours (*P*s < 0.05). Following the protein meal, TG levels showed no significant change during the first 2 hours (*P*s > 0.05) but gradually increased from 3 hours onward (*P*s < 0.05), but the increase was modest. No significant change was observed in TG levels after the glucose meal (*P*s > 0.05). Following the fructose meal, TG levels showed a slight initial reduction (*P*s < 0.05), and did not differ significantly from baseline at 3 and 4 hours (*P*s > 0.05) (see **Figure 2**; **Table 4**). Changes in TC, LDL-C, and HDL-C levels after the different meals were relatively small (see **Figure 2**).

**Table 4:** Change in TG levels after different meals.

TG	High fat	Fat	Protein	Glucose	Fructose	F	*P*
0h	1.32 ± 0.28	1.35 ± 0.28	1.18 ± 0.27	1.17 ± 0.23	1.20 ± 0.46	0.836	0.511
0.5h				1.19 ± 0.31	1.07 ± 0.39^*^	0.706	0.421
1h	1.63 ± 0.46^*^	1.61 ± 0.36^*^	1.20 ± 0.36	1.13 ± 0.33	0.95 ± 0.33^*#^	8.042	<0.001
2h	2.19 ± 0.28^*a^	2.55 ± 0.46^*a^	1.23 ± 0.48	1.06 ± 0.34	0.94 ± 0.27^*^	29.697	<0.001
3h	2.33 ± 0.55^*a^	2.81 ± 0.90^*a^	1.31 ± 0.47^b^	1.01 ± 0.36	1.03 ± 0.26^b^	26.678	<0.001
4h	2.16 ± 0.35^*a^	2.60 ± 0.98^*a^	1.44 ± 0.56^abc^		1.08 ± 0.28^ab^	12.938	<0.001
5h	2.09 ± 0.37^*a^	2.43 ± 0.74^*ac^	1.52 ± 0.49^*abcd^			5.366	0.014
6h	1.91 ± 0.57^*c^	2.07 ± 0.56^*bcde^	1.48 ± 0.43^*ab^			3.892	0.037
F	11.642	18.590	6.022	1.925	5.302		
*P*	<0.001	<0.001	<0.001	0.125	<0.001		

*P<0.05 versus the 0h TG; #P<0.05 versus the 0.5h TG; a, P<0.05 versus the 1h TG; b, P<0.05 versus the 2h TG; c, P<0.05 versus the 3h TG; d, P<0.05 versus the 4h TG; e, P<0.05 versus the 5h TG.

### 3.4 FFA changes after different meals in participants

**Figure 3:**
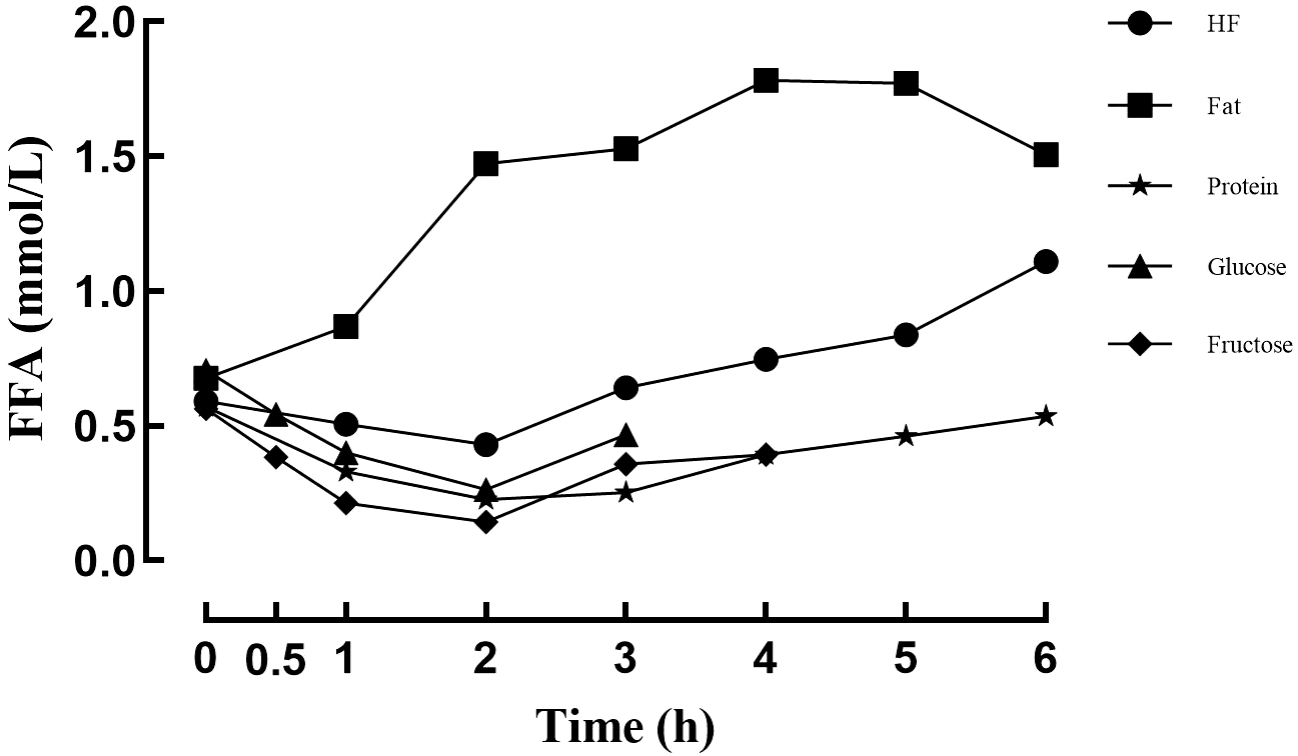
Changes in FFA levels after different meals.

After the high-fat meal, FFA levels declined during the early post-meal period, reached a nadir at 2 hours, and subsequently increased, exceeding baseline levels by 6 hours (*P*s < 0.05). In contrast, the fat meal was accompanied by a gradual rise in FFA levels, with a peak at 4 hours, followed by a decline; however, FFA concentrations remained above baseline at 6 hours (*P*s < 0.05). Following the protein meal, FFA levels decreased initially, reached the lowest point at 2 hours (*P*s < 0.05), and returned to values comparable to baseline at 5–6 hours (*P* > 0.05). After the glucose meal, FFA levels showed an early decrease with a minimum at 2 hours, after which they increased slightly but remained below baseline throughout the rest of the period (*P*s < 0.05). A similar pattern was observed after the fructose meal, with FFA levels reaching a nadir at 2 hours and remaining below baseline throughout the post-meal period (*P*s < 0.05) (see **Figure 3**; **Table 5**).

**Table 5:** FFA levels at different time points after different meals.

FFA	High fat	Fat	Protein	Glucose	Fructose	F	*P*
0h	0.59 ± 0.19	0.68 ± 0.20	0.57 ± 0.10	0.71 ± 0.17	0.56 ± 0.20	1.383	0.257
0.5h				0.54 ± 0.16^*^	0.38 ± 0.14^*^	5.253	0.045
1h	0.51 ± 0.14	0.87 ± 0.15^*^	0.33 ± 0.06^*a^	0.40 ± 0.10^*#^	0.21 ± 0.10^*#^	51.611	<0.001
2h	0.43 ± 0.120^*^	1.47 ± 0.37^*a^	0.23 ± 0.07^*a^	0.26 ± 0.06^*#a^	0.14 ± 0.06^*#a^	108.202	<0.001
3h	0.64 ± 0.19^b^	1.53 ± 0.33^*a^	0.25 ± 0.10^*^	0.47 ± 0.24^*b^	0.36 ± 0.19^*ab^	62.913	<0.001
4h	0.75 ± 0.27^abc^	1.78 ± 0.48^*a^	0.39 ± 0.15^*bc^		0.39 ± 0.14^*ab^	72.923	<0.001
5h	0.84 ± 0.33^abc^	1.77 ± 0.54^*ab^	0.46 ± 0.23^bc^			41.619	<0.001
6h	1.11 ± 0.39^*abcde^	1.51 ± 0.57^*ad^	0.54 ± 0.18^abcd^			28.151	<0.001
F	16.062	23.023	14.079	14.266	20.382		
*P*	<0.001	<0.001	<0.001	<0.001	<0.001		

*P<0.05 versus the 0h FFA; #P<0.05 versus the 0.5h FFA; a, P<0.05 versus the 1h FFA; b, P<0.05 versus the 2h FFA; c, P<0.05 versus the 3h FFA; d, P<0.05 versus the 4h FFA; e, P<0.05 versus the 5h FFA.

### 3.5 Cortisol levels in participants after cconsuming different meals

#### 3.5.1 Cortisol changes after different meals in participants

**Figure 4:**
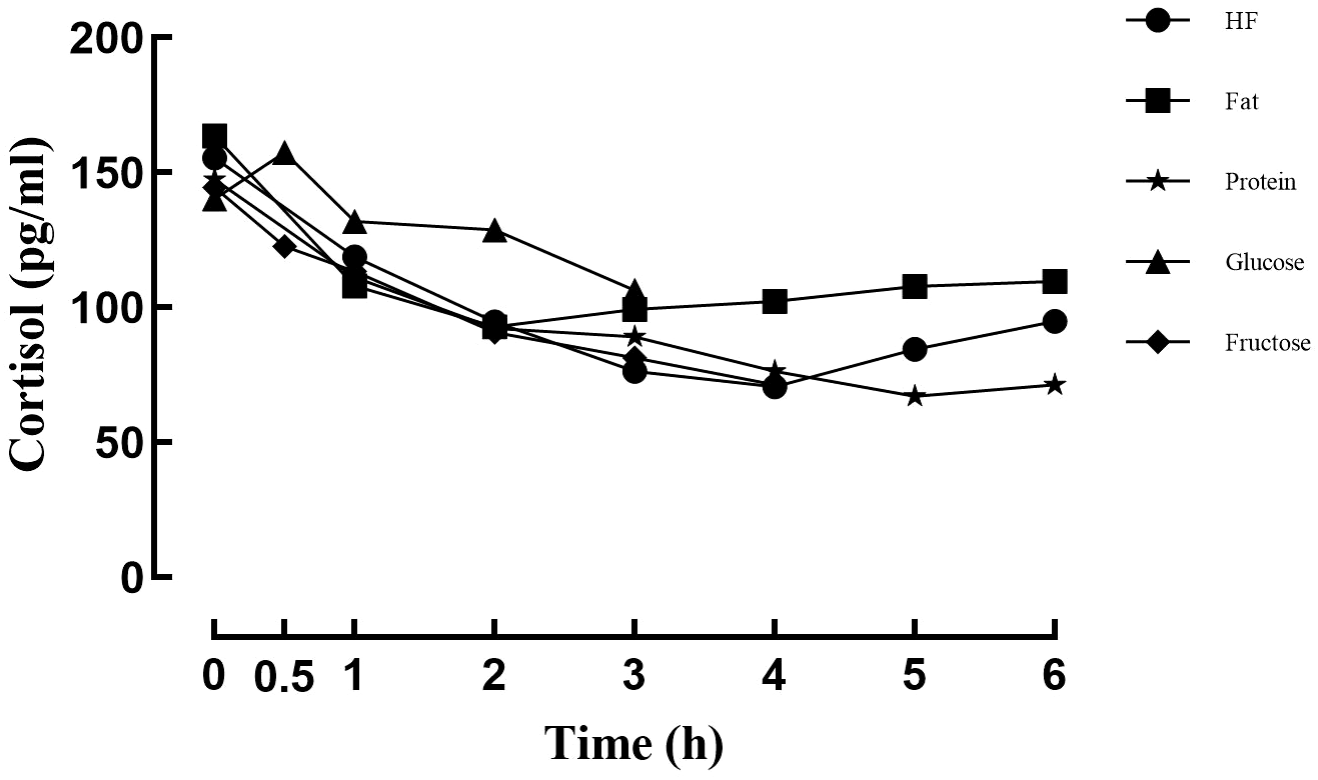
Changes in cortisol levels after different meals.

After consumption of the high-fat meal, cortisol levels initially decreased and then increased, reaching the lowest point at 4 hours. Cortisol levels remained below baseline levels throughout the post-meal period (*P*s < 0.05). Following the fat meal, cortisol levels also showed an initial decrease followed by an increase, with the nadir occurring at 2 hours. Cortisol levels remained below baseline levels (*P*s < 0.05), and no significant differences were observed between 3 to 6 hours after the meal (*P*s > 0.05). After the protein meal, cortisol levels exhibited a continuous downward trend, reaching the lowest level at 5 hours. Cortisol concentrations remained lower than baseline throughout the post-meal period (*P*s < 0.05). Following the glucose meal, cortisol levels initially increased and then decreased, peaking at 0.5 hours post-meal (*P* < 0.05), before declining to below baseline at 3 hours post-meal (P < 0.05). After consumption of the fructose meal, cortisol levels showed a sustained decrease throughout the postprandial period (*P*s < 0.05) (see **Figure 4**; **Table 6**).

**Table 6:** Cortisol levels at different time points after different meals.

Cortisol	High fat	Fat	Protein	Glucose	Fructose	F	*P*
0h	155.51 ± 31.74	163.67 ± 21.57	147.52 ± 36.67	140.26 ± 23.91	144.56 ± 37.84	0.971	0.434
0.5h				157.44 ± 35.48^*^	122.63 ± 43.86^*^	2.995	0.114
1h	118.73 ± 37.47^*^	108.12 ± 28.12^*^	111.11 ± 30.71^*^	131.96 ± 34.08^#^	113.42 ± 44.04^*#^	0.790	0.539
2h	94.66 ± 31.17^*a^	92.79 ± 35.97^*a^	92.28 ± 22.13^*^	128.84 ± 43.09^#^	90.77 ± 33.35^*#a^	2.218	0.084
3h	76.48 ± 20.84^*ab^	99.31 ± 17.28^*^	89.14 ± 29.70^*^	106.44 ± 22.98^*#ab^	81.32 ± 28.11^*#^	3.336	0.019
4h	70.71 ± 11.91^*ab^	102.39 ± 31.35^*^	76.45 ± 26.45^*a^		71.46 ± 33.90^*#a^	3.268	0.035
5h	84.58 ± 20.57^*ad^	107.87 ± 28.26^*^	67.10 ± 26.94^*ab^			7.131	0.005
6h	94.82 ± 15.51^*acde^	109.79 ± 16.42^*^	71.31 ± 30.97^*ab^			10.916	<0.001
F	33.312	13.570	15.991	8.251	12.422		
*P*	<0.001	<0.001	<0.001	<0.001	<0.001		

*P<0.05 versus the 0h cortisol; #P<0.05 versus the 0.5h cortisol; a, P<0.05 versus the 1h cortisol; b, P<0.05 versus the 2h cortisol; c, P<0.05 versus the 3h cortisol; d, P<0.05 versus the 4h cortisol; e, P<0.05 versus the 5h cortisol.

#### 3.5.2 Comparison of cortisol levels after high-fat meal and fat meal intake

A mixed-effects model was used to compare cortisol levels after high-fat meal and fat meal intake, with the participants as the random effect, and meal type, time, and the interaction between meal type and time as the fixed effects. The results indicated that time had a significant negative impact on cortisol levels (P < 0.001). There was no statistically significant difference in cortisol levels before meal intake (P = 0.986). There was an interaction between meal type and time. The rate of cortisol decline in participants after consuming the fat meal decreased more slowly than that after consuming the high-fat meal (P < 0.001) (see **Table 7**).

**Table 7:** Comparison of cortisol levels in participants after consuming high-fat meal and fat meal.

Item	Estimate	Std. error	*P*
Intercept	128.748	7.245	<0.001
Fat	-0.413	10.246	0.986
Slope	-9.798	1.546	<0.001
Interaction	4.349	2.186	0.049

### 3.6 Correlation between cortisol and FFA levels after different meals

The correlations between cortisol and FFA levels at corresponding time points after different meals are presented in **Table 8**. After the high-fat meal, cortisol and FFA levels were positively correlated at 5 hours (r = 0.610, *P* = 0.046) and 6 hours (r = 0.824, *P* = 0.002). Following the fat meal, significant positive correlations were observed at 3 hours (r = 0.632, *P* = 0.037), 4 hours (r = 0.673, *P* = 0.023), and 6 hours post-meal (r = 0.614, *P* = 0.045).

**Table 8:** Univariate linear regression analysis of the correlation between postprandial cortisol and FFA levels.

700 kcal high-fat mixed meal		B	β	t	*P*	Ajusted R^2^
0h FFA	0h Cortisol	77.039	0.453	1.524	0.162	0.117
1h FFA	1h Cortisol	54.715	0.202	0.619	0.551	-0.066
2h FFA	2h Cortisol	96.524	0.370	1.193	0.263	0.041
3h FFA	3h Cortisol	6.548	0.058	0.175	0.865	-0.107
4h FFA	4h Cortisol	18.907	0.427	1.415	0.191	0.091
5h FFA	5h Cortisol	38.328	0.610	2.307	0.046	0.302
6h FFA	6h Cortisol	32.674	0.824	4.359	0.002	0.643
75 g fat meal						
0h FFA	0h Cortisol	30.267	0.286	0.897	0.393	-0.020
1h FFA	1h Cortisol	3.774	0.020	0.060	0.954	-0.111
2h FFA	2h Cortisol	11.856	0.123	0.370	0.720	-0.094
3h FFA	3h Cortisol	33.549	0.632	2.446	0.037	0.332
4h FFA	4h Cortisol	43.736	0.673	2.733	0.023	0.393
5h FFA	5h Cortisol	15.020	0.290	0.908	0.388	-0.018
6h FFA	6h Cortisol	17.835	0.614	2.332	0.045	0.307
75 g protein meal						
0h FFA	0h Cortisol	31.197	0.087	0.263	0.798	-0.103
1h FFA	1h Cortisol	-66.585	-0.124	-0.375	0.716	-0.094
2h FFA	2h Cortisol	42.364	0.133	0.402	0.697	-0.092
3h FFA	3h Cortisol	136.396	0.473	1.609	0.142	0.137
4h FFA	4h Cortisol	9.875	0.057	0.170	0.869	-0.108
5h FFA	5h Cortisol	-29.446	-0.251	-0.777	0.457	-0.041
6h FFA	6h Cortisol	-24.419	-0.139	-0.420	0.684	-0.090
75 g glucose meal						
0h FFA	0h Cortisol	66.512	0.472	1.606	0.143	0.136
0.5h FFA	0.5h Cortisol	5.847	0.027	0.080	0.938	-0.110
1h FFA	1h Cortisol	-68.078	-0.209	-0.643	0.537	-0.062
2h FFA	2h Cortisol	76.714	0.115	0.347	0.737	-0.096
3h FFA	3h Cortisol	54.470	0.559	2.021	0.074	0.236
75g fructose meal						
0h FFA	0h Cortisol	-61.778	-0.334	-1.063	0.315	0.013
0.5h FFA	0.5h Cortisol	55.524	0.178	0.543	0.601	-0.076
1h FFA	1h Cortisol	-60.794	-0.134	-0.404	0.695	-0.091
2h FFA	2h Cortisol	136.147	0.228	0.701	0.501	-0.054
3h FFA	3h Cortisol	26.960	0.178	0.542	0.601	-0.076
4h FFA	4h Cortisol	-10.773	-0.044	-0.133	0.897	-0.109

## 4 Discussion

Both elevated FFA and cortisol levels can mediate a range of adverse metabolic effects, but the relationship between the two remains unclear. In this study, 11 healthy male participants consumed high-fat mixed meals, fat meals, protein meals, glucose meals, and fructose meals. We found that FFA levels were highest after the fat meal, initially increasing and then decreasing. For the other four meals, FFA levels first decreased and then increased, with only the high-fat meal causing FFA levels to rise above baseline. After consuming the high-fat and fat meals, cortisol levels first decreased and then increased, while cortisol levels after the protein and fructose meals showed a declining trend. After the glucose meal, cortisol levels initially increased and then decreased. There were significant positive correlations between cortisol and FFA levels at 5 and 6 hours after the high-fat meal, and at 3, 4, and 6 hours after the fat meal. These findings not only allow us to observe the variation patterns of FFA and cortisol levels after different meals, but they also suggest that the changes in FFA levels after consuming high-fat meals and fat meals for breakfast are closely related to the changes in serum cortisol.

During fasting, adipose tissue releases FFA into the plasma to supply energy to high-demand tissues, such as skeletal muscles and the heart. Postprandial plasma FFA concentrations reflect the balance of lipolysis within adipose tissue, both intracellular and extracellular. In contrast to the increase in plasma triglyceride (TG) levels following mixed meals, plasma FFA levels initially decrease sharply and subsequently rise. This initial decline is primarily attributed to the regulation of intracellular lipoprotein lipase (LPL) activity by insulin (9–12). LPL is a key enzyme in lipid metabolism, regulated by insulin. It hydrolyzes TGs found in chylomicrons and very-low-density lipoproteins (VLDL), with a higher affinity for chylomicron-TGs. This process releases FFAs, which are then stored or utilized in adipose and muscle tissues. LPL thus plays a critical role in the distribution of dietary FFAs (13, 14). Insulin is a potent inhibitor of lipolysis and acts as the primary regulator of postprandial FFA metabolism. Plasma FFA levels are nearly inversely proportional to insulin concentrations. After consuming carbohydrate-rich meals, insulin levels rise, suppressing lipolysis and shifting tissue metabolism from FFA release to storage, thereby lowering FFA levels. As insulin levels return to baseline in the later postprandial phase, lipolysis resumes (15). Moreover, not all FFAs released during chylomicron-TG hydrolysis by LPL are absorbed; some spill over into the plasma, causing a rebound increase in FFA levels. This spillover can account for 40-50% of total postprandial plasma FFA concentrations (16). This study’s findings align with previous literature. After high-fat mixed meals, protein meals, glucose meals, and fructose meals, participants showed initial insulin elevation and a corresponding decrease in FFA levels. However, approximately three hours postprandially, as insulin concentrations approached baseline, FFA levels rose significantly compared to the second hour. Only high-fat meals led to FFA levels exceeding baseline in the later postprandial phase, likely due to the specific effects of high-fat diets. For fat-only meals, insulin fluctuations were minimal, and FFA levels gradually rose alongside chylomicron-TG concentrations before declining in the later postprandial phase. Consistent with these findings, research by Hernandez TL et al. suggested that the ratio of dietary fat to carbohydrates influences postprandial FFA levels, with higher fat proportions preventing the initial decline in FFAs (17). While some studies have reported that FFA levels may slightly exceed baseline 5-6 hours after consuming 75 grams of glucose or fructose (18), this is likely due to lipolysis rather than dietary intake. Glucose and fructose minimally contribute to intestinal FFA levels, and in healthy individuals, OGTT show a return to baseline blood glucose levels within three hours. Glucose is fully absorbed by then, and fructose absorption occurs even faster (19). Considering that the slight increase in FFA is caused by fat breakdown and has little relation to food intake, in order to reduce the number of blood draws for the subjects, the blood draw for the glucose meal and fructose meal tests is conducted until 3-4 hours after the meal.

Normal plasma cortisol levels exhibit a distinct diurnal rhythm, peaking within one hour after waking and gradually decreasing throughout the day. Cortisol levels begin to rise around 4 AM. An important characteristic of cortisol is its increase after meals. As early as 1979, researchers observed that plasma cortisol levels rise after eating, particularly after lunch, with peak levels typically occurring within one hour post-meal (20, 21). However, previous literature often focused on cortisol levels within 3 hours after meals, and research on the longer-term postprandial changes in cortisol levels is limited. Research by Martens MJ et al. suggested that after consuming carbohydrate-only foods (such as dextrin and maltose), cortisol levels rise, reaching a peak at 0.5 hours post-meal (22). In this study, after consuming glucose, cortisol levels first increased and then decreased, peaking at 0.5 hours post-meal, consistent with previous reports. However, after consuming the other meals, no immediate peak in cortisol levels was observed. This may be due to the timing of the experiment, which was conducted between 7:00 and 8:00 am, a period when cortisol levels are already decreasing after reaching their peak in the early morning. Future studies could consider starting the experiments around lunchtime to minimize the impact of the circadian rhythm on cortisol levels. Another factor could be the longer intervals between blood draws in this study, which limited the ability to capture the changes in cortisol levels within the first hour after meals. Increasing the frequency of blood draws during this period could provide more insight into cortisol fluctuations immediately following meals.

11β-Hydroxysteroid dehydrogenase type 1 (11β-HSD1) is a cortisol-metabolizing enzyme that primarily converts inactive cortisone into its active form, cortisol, thereby regulating its local concentration in tissues and organs. This enzyme acts as a “local amplifier” for glucocorticoids (GCs) (23). FFA induce endoplasmic reticulum stress in various peripheral tissues and cells, which in turn promotes the translation of the metabolic regulator CCAAT/enhancer-binding protein α (C/EBPα) (24, 25). C/EBPα is an effective transcriptional activator of the 11β-HSD1 gene (26). Previous studies have suggested that elevated FFA levels can lead to an acute increase in the activity of 11β-HSD1 in adipose tissue, potentially further increasing cortisol concentrations (27, 28). In this study, following the consumption of high-fat and fatty meals, cortisol levels initially decreased due to circadian rhythm effects. However, as FFA levels increased later postprandially, cortisol levels also increased. Significant positive correlations were observed between cortisol and FFA levels at 5-6 hours post-high-fat meal and at 3, 4, and 6 hours after consuming the fatty meal. This suggests that the changes in FFA levels after consuming high-fat meals and fat meals for breakfast are closely related to the changes in serum cortisol. Although cortisol levels increased with FFA levels following the fat meal, no significant differences in cortisol levels were observed between 2-6 hours post-meal, indicating that cortisol does not increase indefinitely and may be influenced by the circadian rhythm’s decline. For protein, glucose, and fructose meals, FFA levels did rise in the later stages post-meal, but the increase was modest, and no significant correlation was found between cortisol and FFA levels. This suggests that the impact of FFA on cortisol levels might be less pronounced when these types of meals are consumed.

High concentrations of FFA and cortisol after meals can cause metabolic abnormalities (29). Firouzi S et al. also suggested that differences in postprandial responses due to dietary components can impact metabolic and vascular parameters (30). The composition of macronutrients in the diet plays a significant role in maintaining good health, and the impact of dietary components on metabolism needs to be taken seriously.

This study is the first to propose that the changes in FFA levels after consuming high-fat meals and fat meals for breakfast are closely related to the changes in serum cortisol. However, this study still has certain limitations. We only included healthy adult male subjects, and the sample size was small. The research results of Anderson GW et al. suggest that the cortisol level in men after a high-cholesterol meal is higher than that in women, while the cortisol level in women is higher after the meal (31). Therefore, gender can affect the degree of increase in cortisol after a meal. The menstrual cycle also affects cortisol levels, especially in women during the late luteal phase, when the serum cortisol level fluctuates greatly (32). Therefore, the subjects included in this study were all male participants. The fat meal, protein meal, glucose meal and fructose meal all had 75g, but the calories of the fat meal were different from those of the other three meals. Further research in the future needs to explore the characteristics of changes in cortisol and FFA levels after consuming meals with the same calorie but different components.

## 5 Summary

With the change of lifestyle, the prevalence of metabolic diseases is increasing and they are becoming younger and younger. As dietary fat content increases, postprandial FFA level also rises. The changes in FFA levels after consuming high-fat meals and fat meals for breakfast are closely related to the changes in serum cortisol.

## Data Availability

The datasets presented in this article are not readily available because Involving patient privacy. Requests to access the datasets should be directed to DL: 15632277371@163.com.
